# Mitochondrial genome of bronze-winged jacana (*Metopidius indicus*, Latham 1790)

**DOI:** 10.1080/23802359.2021.1945971

**Published:** 2021-08-01

**Authors:** Jitmat Thintip, Syed Farhan Ahmad, Worapong Singchat, Nararat Laopichienpong, Aorarat Suntronpong, Thitipong Panthum, Dung Ho My Nguyen, Nattakan Ariyaraphong, Narongrit Muangmai, Warong Suksawet, Prateep Duengkae, Kornsorn Srikulnath

**Affiliations:** a Laboratory of Animal Cytogenetics and Comparative Genomics (ACCG), Department of Genetics, Faculty of Science, Kasetsart University, 50 Ngamwongwan, Chatuchak, Bangkok, Thailand; b Special Research Unit for Wildlife Genomics (SRUWG), Department of Forest Biology, Faculty of Forestry, Kasetsart University, 50 Ngamwongwan, Chatuchak, Bangkok, Thailand; c Animal Genomics and Bioresource Research Center (AGB Research Center), Faculty of Science, Kasetsart University, 50 Ngamwongwan, Chatuchak, Bangkok, Thailand; d The International Undergraduate Program in Bioscience and Technology, Faculty of Science, Kasetsart University, 50 Ngamwongwan, Chatuchak, Bangkok, Thailand; e Department of Fishery Biology, Faculty of Fisheries, Kasetsart University, 50 Ngamwongwan, Chatuchak, Bangkok 10900, Thailand; f Center for Advanced Studies in Tropical Natural Resources, National Research University-Kasetsart University, Kasetsart University, Bangkok, Thailand (CAST NAR, NRU-KU, Thailand); g Center of Excellence on Agricultural Biotechnology: (AG-BIO/MHESI), Bangkok, Thailand

**Keywords:** Metopidius, next-generation sequencing, mitogenome, phylogenetics

## Abstract

We reported the mitochondrial genome (mitogenome) of bronze-winged jacana (*Metopidius indicus*, Latham 1790). The circular mitogenome was 17,208 base pairs (bp) in length, containing 13 protein-coding genes, two rRNAs, 22 tRNAs, and a non-coding control region. A DNA spacer 109 bp long was also detected between *ND5* and *Cytb*. Phylogenetic analysis indicated that *M. indicus* was more closely related with the genera *Himantopus*, *Jacana* and *Hydrophasianus*. This annotated mitogenome reference can be utilized as a data resource for comparative mitogenomics of waders or shorebirds, with possible use in ecological and evolutionary studies.

The bronze-winged jacana (*Metopidius indicus*, Latham 1790) is a wader in the family Jacanidae. It is an ideal species for the study of polyandry (Miller et al. 2016) and widely distributed across the Indian subcontinent and Southeast Asia (Ali and Ripley 1980; IUCN 2013). The bronze-winged jacana shows greater reversed sexual size dimorphism than any other group of birds (Jenni 1996). The genus *Metopidius* was introduced by the German zoologist Johann Georg Wagler in 1832 (Wagler 1832). According to the World Bird List Version 9.2. International Ornithologists’ Union, *Metopidius indicus* is the only species within the genus (Avibase 2019; Frank and David 2019); however, the genetic features of this species have not been previously studied. Here, a complete mitochondrial genome of *M. indicus* was determined. The bronze-winged jacana specimen was collected as a carcass found at Kasetsart University, Bangkok, Thailand (13.847331°N, 100.572067°E). Animal ethics were submitted to the supervisory committee of Kasetsart University (ACKU63-SCI-022) and the specimen was stored in the Thailand Natural History Museum (No. THM21090; Contact person: Sunchai Makchai, E-mail: sunchai@nsm.or.th). Whole genomic DNA was extracted from the liver using the standard salting-out protocol (Supikamolseni et al. 2015). Next-generation sequencing was performed using an Illumina HiSeq3000 platform at Vishuo Biomedical Ltd., Bangkok, Thailand, with a coverage of 50×. The quality of Illumina reads was evaluated with FastQC and the raw reads were trimmed to discard adapters using Trimmomatic software V0.32 (Bolger et al. 2014). The cleaned reads were then assembled to construct the mtgenome using MitoZ version_2.4-alpha (Meng et al. 2019). We set the multi-Kmer mode, ‘–genetic_code 2,’ ‘–clade chordata’ in MitoZ pipeline to identify the mitogenomic sequences, de novo assembly and annotations. Complete mitogenome sequences consisted of 17,208 bp for bronze-winged jacana (GenBank Accession no. MW482963, SRA: SSR13451235, BioProject: PRJNA692351), comprising 13 protein-coding genes, 22 tRNAs, 2 rRNAs, and a control region, similar to the mitogenome structure of birds (Desjardins and Morais 1990). Non-coding spacers were detected with 109 bp between *ND5* and *Cytb*, which differed from other species in the family Jacanidae. Overall AT content value for the *M. indicus* mitogenome was 53.7%, with an average nucleotide diversity of 0.190 ± 0.002. The methodology of community phylogenetics was used to compare the phylogenetic dispersion of Charadriiformes. A phylogenetic tree was constructed based on twelve concatenated protein-coding genes of 21 species from Charadriiformes and Galliformes, using Bayesian inference with MrBayes version 3.2.6. (Huelsenbeck and Ronquist 2001). *ND6* sequences were excluded in the phylogenetic analysis because of high bias toward transitions reported from previous tests of substitution models (Duchêne et al. 2011). Phylogenetic analysis of bronze-winged jacana showed an ancestral relationship with genera *Himantopus*, *Jacana* and *Hydrophasianus*, corresponding to a similar tree structure derived from TimeTree (Figure 1). These complete mitogenomes will allow the creation of a reference annotated genome, and provide valuable information at the molecular level that can be utilized to sustain conservation management of the bronze-winged jacana.

**Figure 1.**
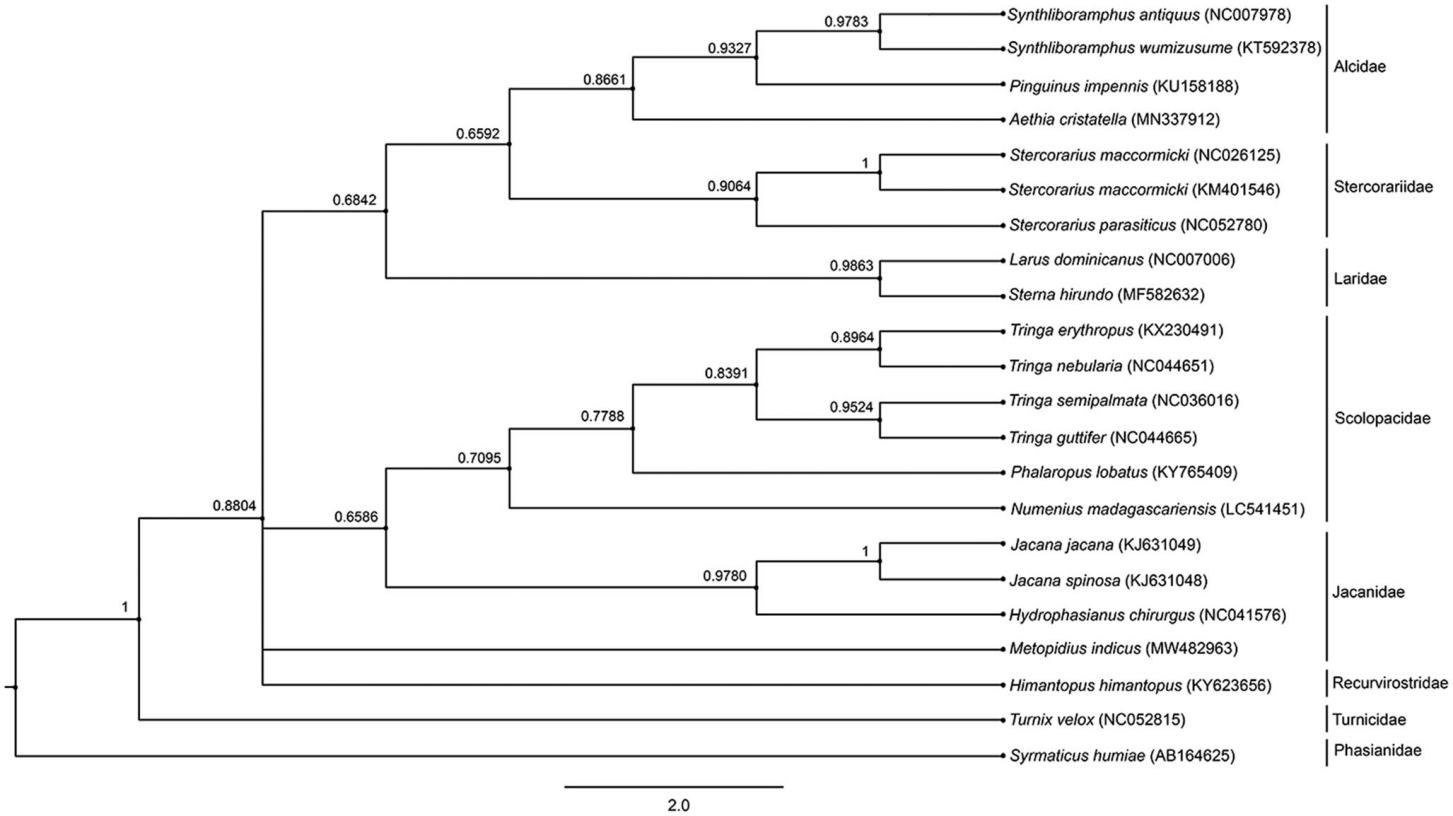
Phylogenetic relationships among 12 concatenated mitochondrial protein-coding genes, without *ND6* sequences of 21 mitochondrial genomes, including *Syrmaticus humiae* as the outgroup, using Bayesian inference analysis. The complete mitochondrial genome sequence was downloaded from GenBank. Accession numbers are indicated in parentheses after the scientific names of each species. Support values at each node are Bayesian posterior probabilities, while branches length represents the number of nucleotide substitutions per site.

## Data Availability

Data supporting the study findings are available in GenBank through the NCBI at https://www.ncbi.nlm.nih.gov. Isolated mitogenome reads were deposited at NCBI SRA database (accession ID: SSR13451235), and the assembled mitogenome sequences are available in GenBank (accession ID: MW482963).
